# Enhanced Production of Erythritol from Glucose by the Newly Obtained UV Mutant *Yarrowia lipolytica* K1UV15

**DOI:** 10.3390/molecules29102187

**Published:** 2024-05-08

**Authors:** Anita Rywińska, Ludwika Tomaszewska-Hetman, Piotr Juszczyk, Magdalena Rakicka-Pustułka, Adam Bogusz, Waldemar Rymowicz

**Affiliations:** Department of Biotechnology and Food Microbiology, Wrocław University of Environmental and Life Sciences, Chełmońskiego Str. 37, 51-630 Wrocław, Poland; anita.rywinska@upwr.edu.pl (A.R.); piotr.juszczyk@upwr.edu.pl (P.J.); magdalena.rakicka-pustulka@upwr.edu.pl (M.R.-P.); waldemar.rymowicz@upwr.edu.pl (W.R.)

**Keywords:** erythritol, glucose, *Yarrowia lipolytica*, fed-batch culture, high biomass

## Abstract

Erythritol is a polyol with a sweet taste but low energy value. Thanks to its valuable properties, as well as growing social awareness and nutritional trends, its popularity is growing rapidly. The aim of this study was to increase the effectiveness of erythritol production from glucose using new UV mutants of the yeast *Yarrowia lipolytica* obtained in the Wratislavia K1 strain. The ability of the new strains to biosynthesize erythritol and utilize this polyol was examined in shake-flask cultures and fed-batch processes conducted in a stirred tank reactor with a total glucose concentration of 300 and 400 g/L. The Wratislavia K1 strain produced erythritol most efficiently (97.5 g/L; 192 h) at an initial glucose concentration of 250 g/L (total: 300 g/L). New strains were assessed under such conditions, and it was noted that the highest erythritol concentration (145 g/L; 183 h) was produced by the K1UV15 strain. A significant improvement in the erythritol biosynthesis efficiency (148 g/L; 150 h) was achieved upon the increase in (NH_4_)_2_SO_4_ to 3.6 g/L. Further, in the culture with such a concentration of the nitrogen source and increased total glucose level (400 g/L), the K1UV15 strain produced 226 g/L of erythritol within 281 h.

## 1. Introduction

Polyhydric alcohols, also known as polyols, are compounds that have more than one hydroxyl group in their molecular structure. This group includes, among others, erythritol, mannitol, arabitol, xylitol, lactitol, and maltitol [1]. Of these, erythritol has been gaining popularity in recent years, especially in the food industry [2,3]. Erythritol is a natural sweetener with 70% of the sweetness of sucrose. In comparison to other polyols, it is characterized by the lowest molecular weight and high derepression of water activity. Its energy value is ≤0.2 kcal/g; therefore, it is classified as a low-calorie compound [4]. The low energy value of erythritol is due to the fact that it is unmetabolized—approximately 80% of erythritol is recovered in urine within 24 h when used at a dose of 1 g/kg of body weight. Moreover, erythritol consumption has no effect on insulin and blood glucose levels [5]. Studies have indicated that it improves glycemic control and vascular function in diabetic patients [6]. When ingested, erythritol provides a cooling sensation in the mouth due to the negative heat of the solution (−23.3 Kcal/g) [7]. The compound is not only safe for tooth enamel but even reduces the formation of dental plaque [8]. Detailed studies performed on dogs, rats, and mice have shown that erythritol has no carcinogenic potential or teratogenic properties [9].

The World Health Organization recognized erythritol as safe in 1987, and in 1997 it received generally recognized as safe (GRAS) status from the United States Food and Drug Administration. In Japan, erythritol has been widely used as a substitute for sucrose since 1990. In the EU, erythritol is currently used as a food additive with the number E968 in accordance with Directive 2006/52/EC of the European Parliament and of the Council of 5 July 2006 on sweeteners used in foodstuffs. Changes in dietary trends observed in recent years favor the spread of erythritol and its increasing use as a sugar substitute in low-calorie foods, baked goods, confectionery, gums, and drinks. Its task is to improve the taste profile and aromaticity of drinks that are free of added sugar [10]. It is also used as a flavor enhancer, stabilizer and thickener, sealant, and texturizer, and as an excipient in pharmaceutical preparations [2,11,12]. It is estimated that the global production of erythritol is currently 60,000 t/year, which is a threefold increase compared to 23,000 t produced in 2011 [1]. In 2023, the global erythritol market is expected to be valued at USD 225.86 million. The total demand for erythritol is projected to grow at a CAGR of 7.6% from 2023 to 2033, reaching approximately USD 449.6 billion by 2033 [13]. The price of erythritol is at a level comparable to other compounds produced in the process of microbial biosynthesis, e.g., glutamic acid or citric acid [14].

Various yeast-like fungi genera are capable of erythritol biosynthesis: *Trigonopsis*, *Torula*, *Trichosporon*, *Trichosporonoides*, *Candida*, *Pichia*, *Aureobasidium*, *Moniliella*, and *Yarrowia* [9]. However, due to lower efficiency, productivity, or the formation of by-products (e.g., ribitol), the production of erythritol using some of them cannot be implemented on a commercial scale [15]. The most commonly used microorganisms in industrial production are *Moniliella pollinis*, *Moniliella megachiliensis*, and, more recently, *Y. lipolytica* [14]. In 2003, the Chinese company Baolingbao Biology Co. began producing erythritol with the use of *Yarrowia* yeast [16]. Recently, this yeast has also been increasingly considered as an attractive producer of sugar alcohols other than erythritol, i.e., mannitol and arabitol [17,18].

Substrates used for the production of erythritol by various groups of microorganisms include glucose or fructose. Most often used as the main source of carbon, glucose (dextrose) is obtained from wheat or corn starch as a result of the chemical or enzymatic hydrolysis of these products. Other substrates used are pure sucrose, as well as molasses or sugar cane juice containing it. However, it should be noted that the application of disaccharides is limited by the inability of individual yeast species to hydrolyze O-glycosidic bonds [9,19]. Studies conducted on the biosynthesis of erythritol by *Y. lipolytica* have shown that glycerol can be successfully used in this process [18,20]. Glycerol became a particularly popular substrate in biotechnological processes between 2000 and 2010 due to the increased production of biodiesel, which resulted in significant surpluses of glycerol in both the EU and the USA. Together with overestimated production forecasts for this biofuel, this led to a decline in glycerol prices from approximately USD 3.2/kg to below USD 0.5/kg in the EU. The current prices of glycerol vary depending on its type and region, but it is generally around USD 0.17/kg [21]. The surplus in glycerol has also spurred the development of new technologies for transforming it into high-value products. Currently, glycerol is primarily used as an intermediate chemical for various products such as cosmetics, food, and pharmaceuticals [22]. In recent years, the price of glucose has been characterized by mild fluctuations and slight declines in both import and export prices. The market report [23] estimates the global industrial glucose market size was approximately USD 40 billion in 2022, indicating significant market demand and its utilization in various industries such as food, pharmaceuticals, and fermentation processes. The market price of glucose for biotechnological applications appears to range from approximately USD 0.10/kg to USD 0.26/kg, which currently makes it a very competitive substrate in relation to glycerol [24,25,26]. However, so far, only limited reports are available on the use of glucose for the production of this polyhydric alcohol using the yeast *Y. lipolytica* [20,27,28,29].

Our previous research indicated that the Wratislavia K1 strain is particularly predisposed to the production of erythritol from glycerol, not only due to good parameters of the biosynthetic process but also because it does not utilize erythritol, a feature not found in wild strains [20]. However, initial research on the production of erythritol from glucose was not satisfactory, which led to a planned process of improving this yeast strain using UV radiation mutagenesis. UV mutagenesis is a relatively easy and quick method that allows for the generation of a large number of mutants with altered metabolic characteristics [30,31]. It should be noted, however, that these changes are random and do not guarantee the improvement of a specific biosynthetic pathway; therefore, the obtained pool of strains must be verified each time in terms of suitability for the production of a specific compound.

The aim of this study was to assess the effectiveness and efficiency of erythritol production from glucose using newly obtained UV mutants of the yeast *Yarrowia lipolytica*.

## 2. Results and Discussion

### 2.1. UV Mutagenesis and Preliminary Characterization of New Strains

**Figure 1:**
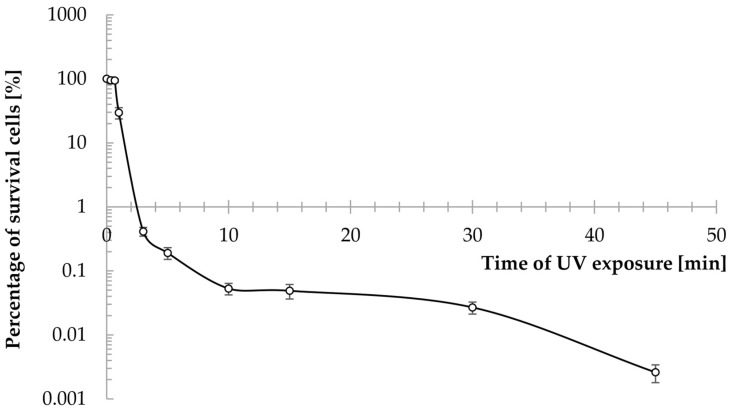
Survival curve of *Y. lipolytica* Wratislavia K1 strain exposed to UV irradiation. The error bars represent the standard deviation of three replications.

The *Y. lipolytica* Wratislavia K1 strain used in the present study is an acetate mutant isolated from the culture of the yeast *Y. lipolytica* Wratislavia 1.31 under chemostat conditions, and its history was described in detail previously by Rywińska et al. [32]. In the first stage of the present study, the Wratislavia K1 strain was subjected to mutagenesis using UV, with the intention of improving the efficiency of the glucose-to-erythritol conversion. The mutagenesis process has been successfully applied before to improve the erythritol biosynthesis process, but new strains were tested only when glycerol was used as the sole source of carbon and energy [33,34]. The effect of exposure time on the survival of the Wratislavia K1 strain is shown in Figure 1. After 1 min of exposure, the population reduction rate was very high: 70% (Figure 1). After 5 min, only about 0.2% of live cells remained in the irradiated suspension. In a similar experiment, conducted for the same strain but using different station parameters and a higher power of lamp, after 20 s of exposure, the population reduction rate was 54.2%. In the case of the Wratislavia 1.31 strain, after 10 s of exposure, the reduction rate was 76% [35], which indicates that the sensitivity to UV radiation depends on the strain and the radiation power. In this study, the mutagenization process was carried out by irradiating cells of the Wratislavia K1 strain for a time, giving a cell survival rate of approximately 0.1% (5, 10, 15, and 30 min) (Figure 1). After mutagenization, eight cultures with various morphologies and colony sizes were isolated and then tested for their ability to grow and the efficiency of erythritol utilization (Table 1 and Table S1).

The lack of erythritol utilization ability is a characteristic feature of the parental strain Wratislavia K1, which was observed for the first time during citric acid biosynthesis with the yeast [36]. Among the acetate mutants tested at that time, only Wratislavia K1 did not utilize the previously produced erythritol, which had a significant impact on the low selectivity of the citric acid production process. On the other hand, this unique feature enables significant improvement to be achieved in the selectivity of the production during the biosynthesis of erythritol. It was already demonstrated that the addition of a nitrogen source at the end of the process mobilizes the cells of the Wratislavia K1 strain to utilize all compounds available in the culture broth as a carbon source, except for erythritol, which leads to the almost complete elimination of by-products [37]. In this study, the assessment of the ability of strains to utilize erythritol was carried out in 3-day shake-flask cultures in a medium containing 43 g/L of erythritol as the only carbon source. After the culture, in the case of the parental strain Wratislavia K1, and mutant strains K1UV1 and K1UV15, no increase in the level of biomass was observed, and the erythritol concentration in the post-culture broth was the same as that introduced initially into the medium. The lowest substrate concentration (20.4 g/L) was determined after the culture of the K1UV11 strain, which means that this strain had the highest erythritol utilization rate, which was 313.9 mg/Lh (Table 1). 

In the next experiment, the biosynthesis of erythritol from glucose was performed in 4-day shake-flask cultures. After this time, the erythritol concentration, depending on the strain, ranged from 20.3 (K1UV16) to 27.4 g/L (K1UV20). It is worth noting that only the K1UV16 strain produced less erythritol than the Wratislavia K1 strain. The production parameters—the yield and specific production rate of erythritol—calculated on the basis of the obtained results, were the lowest for the K1UV16 strain and the highest for K1UV20 and K1UV1. Interestingly, it was noted that the two UV mutants K1UV1 and K1UV15, characterized by the lack of erythritol utilization and, additionally, K1UV18, also stood out in terms of the high glucose utilization rate (Table 1). The characteristics of the strains in the above-mentioned experiments indicated that most of the new strains utilized glucose faster, producing more erythritol than the initial strain, but only two strains, similar to the parental strain Wratislavia K1, did not utilize erythritol. Since the results did not clearly indicate which strain was the best, all strains were subjected to further technological characterization on a larger scale in bioreactor cultures.

### 2.2. Selection of the Initial Glucose Concentration for Erythritol Biosynthesis

**Figure 2:**
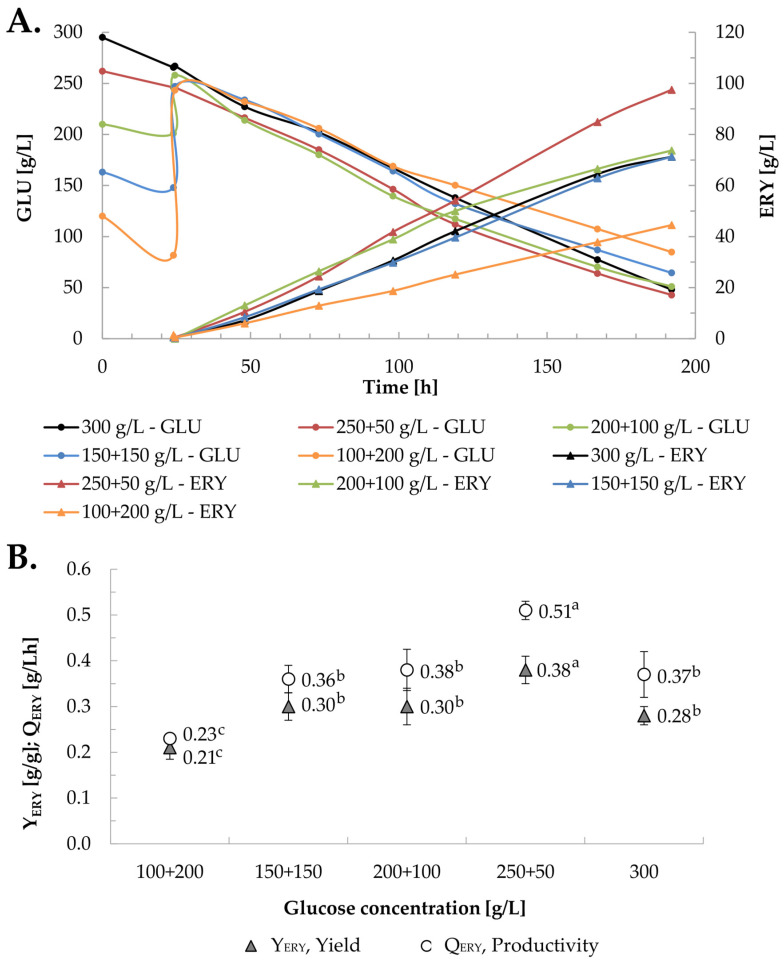
Effect of initial glucose concentration on the course of erythritol biosynthesis (**A**) and erythritol yield (Y_ERY_) and productivity (Q_ERY_) (**B**) by *Y. lipolytica* Wratislavia K1 strain in bioreactor batch and fed-batch cultures. Production medium contained 2.6 g/L of (NH_4_)_2_SO_4_. Values of the same parameter marked with different letters differ significantly at *p* ≤ 0.05.

The overproduction of erythritol on the part of yeast and fungi is a response of these microorganisms to environmental hyperosmotic or oxidative stress. Therefore, the high osmotic pressure of the culture medium is a necessary condition for the production of erythritol, which has been studied in various aspects in the case of the parent strain Wratislavia K1 in cultures with glycerol, but no studies have been carried out on the correlation between osmotic pressure and glucose concentration in relation to the biosynthesis of erythritol from this sugar. Therefore, in the next stage of this research, the influence of the initial glucose concentration on the parameters of erythritol biosynthesis was examined for the parent strain Wratislavia K1, which was intended to indicate the production conditions for the cultivation of the mutants tested in this study (Figure 2). During cultivation, increased osmotic pressure can be obtained by adding salt to the medium or using a high concentration of substrate [18,38]. In *Y. lipolytica* yeast cultures carried out in a medium with glycerol and corn steep liquor, the addition of NaCl, KCl, and CaCl_2_ was tested at concentrations that allowed us to obtain an initial osmotic pressure of the medium of approximately 3.3 Osm/kg. Compared to the culture without salt, under such conditions, a significant increase in the activity of enzymes key to the overproduction of erythritol was observed—a 2.5-fold increase in the case of transketolase and up to a 5-fold increase in erythrose reductase [39]. The presence of 32.5 g/L of NaCl in the mineral medium with 150 g/L of glycerol generated an osmotic pressure of 2.9–2.8 and 2.1–1.9 Osm/kg, respectively, in the growth phase and the stationary phase. As a result, the production of erythritol in the cultures with salt, depending on the purity of the glycerol used (pure or waste) and pH (3.0 or 4.5), was higher by between 9 and 37% compared to cultures without NaCl [40]. Xiaoyan et al. [41] tested the possibility of using waste cooking oil for the biosynthesis of erythritol. In the medium containing 30 g/L of this substrate, they checked the addition of NaCl from 0 to 100 g/L, which caused an increase in osmotic pressure from 0.08 to 3.43 Osm/L. Under such conditions, the most erythritol was produced in the culture carried out in the medium containing 80 g/L of NaCl. Previous research [27] and the results of this study (Figure 2) confirmed that high osmotic pressure generated by adding another portion of substrate to the culture after 24 h, to the grown biomass, will also result in high erythritol production. In this study, five production processes were carried out, starting at 100, 150, 200, 250, and 300 g/L of glucose (Figure 2A), which generated an initial osmotic pressure from 0.9 to 1.7 Osm/kg. In order to obtain a total glucose concentration of 300 g/L, the remaining part of the substrate was added after 24 h. Depending on the dosed glucose portion, the addition of substrate caused an increase in the osmotic pressure of the culture broth by up to 0.8 Osm/kg. The cultures were conducted for 192 h, after which, depending on the breeding variant, the residual glucose concentration ranged from 42.8 to 84.8 g/L, while the amount of erythritol produced ranged from 44.5 to 97.5 g/L (Figure 2A). The highest values of erythritol production parameters obtained using the Wratislavia K1 strain were observed in the 250 + 50 g/L culture variant (Figure 2B). Notably, the erythritol concentration was high and reached 97.5 g/L (Figure 2A) even though no NaCl was added to the medium. In this culture, the osmotic pressure varied from 1.5 to 1.8 Osm/kg. The erythritol production yield was 0.38 g/g, the volumetric productivity reached 0.51 g/Lh, and the selectivity was 82% (Figure 2B). Rakicka et al. [27] compared the production of erythritol from glycerol, inulin, and fructose by transformants with the expression of the inulinase gene in fed-batch cultures. The yeast was grown in a medium containing 40 g/L of glycerol, inulin, glucose, or fructose, and then 200 g/L of glycerol was added after 24 h. The production yield of this polyol ranged from 0.38 to 0.6 g/g, while the volumetric production rate varied from 0.89 to 1.3 g/Lh, depending on the kind of initial substrate and strain. For the Wratislavia K1 strain, when glucose was used at the beginning of the culture, the erythritol production yield was 0.43 g/g, whereas the selectivity reached 78% [27]; therefore, the obtained parameters were comparable to the present results.

### 2.3. Erythritol Production Using UV Mutants

In the next stage of the research, eight new strains were tested using the substrate dosing strategy of 250 + 50 g/L, previously found to be the most effective (Table 2). The cultures were conducted until the complete depletion of glucose, which lasted from 145 to 222 h, depending on the strain. The erythritol biosynthesis parameters obtained for six of the new mutants were higher than those of the parent strain which produced 112 g/L of erythritol with a yield of 0.37 g/g and a productivity of 0.5 g/Lh. For comparison, in a batch culture with continuous substrate feeding, in which a total concentration of 300 g/L of glycerol was used, the yield and volumetric production rate of erythritol biosynthesis were higher: 0.56 g/g and 1.00 g/Lh, respectively [20]. In the present study, the highest yield, volumetric production rate, and selectivity of erythritol production values were observed for the K1UV15 strain. It should also be emphasized that in the culture with this strain, no foam formation was observed throughout the production process, which is a very desirable feature in the technological process, especially on an industrial scale, which is why this strain was recommended for the next stage of research.

### 2.4. Effect of (NH_4_)_2_SO_4_ Concentration on Erythritol Production by K1UV15 Strain

**Figure 3:**
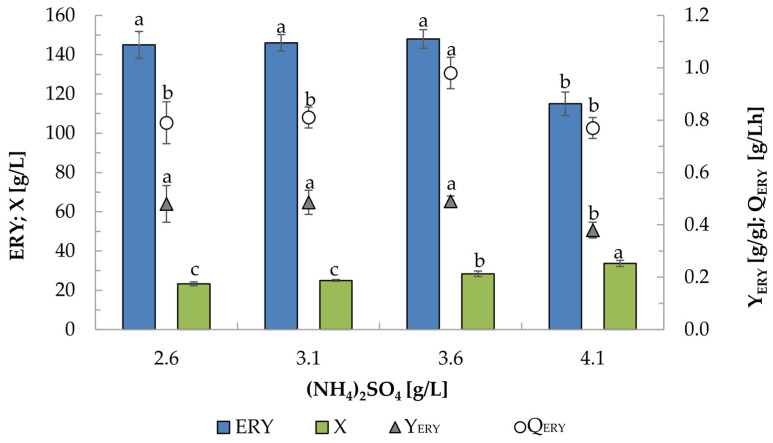
The impact of (NH_4_)_2_SO_4_ (ammonium sulfate) concentration on the production of erythritol by the K1UV15 mutant strain of *Y. lipolytica* in fed-batch culture. Culture conditions: 250 + 50 g/L glucose dosing variant. Y_ERY_—yield of erythritol in terms of substrate consumed; Q_ERY_—erythritol volumetric production rate [g/Lh]. Values of the same parameter marked with different letters differ significantly at *p* ≤ 0.05. The error bars represent the standard deviation of two replications.

In the next experiment, the effect of the nitrogen source concentration on the erythritol biosynthesis process by the selected K1UV15 strain was examined (Figure 3). In this study, all cultures were carried out using an inorganic salt, (NH_4_)_2_SO_4_, which was previously identified as the best source of nitrogen for the biosynthesis of erythritol from glycerol by the Wratislavia K1 strain [42]. Most authors recommend organic compounds as a source of nitrogen for the process of erythritol biosynthesis. Most often, this compound is yeast extract, corn steep powder, or liquor, but maltose extract, casamino acid, beef extract, peptone, tryptone, and soybean flour have also been examined [28,29,43,44]. In a medium containing only glycerol, and yeast extract, with or without NaCl, the Wratislavia K1 strain exhibited very good erythritol production parameters—a yield in the range of 0.54–0.62 g/g and a productivity of 0.61–1.2 g/Lh [37]. The values of these parameters depended primarily on the concentration of biomass in the production process, which, depending on the purity of the substrate and yeast extract concentration, ranged from 16.7 to 41.8 g/L. It should be noted, however, that the application of an organic nitrogen source, such as yeast extract, in the production medium may affect the color of erythritol crystals formed after the crystallization process, necessitating an additional purification procedure. For this reason, (NH_4_)_2_SO_4_ was used in this study as a source of nitrogen and was tested at different concentrations (Figure 3). It was observed that with the increase in the (NH_4_)_2_SO_4_ concentration, the biomass level increased from 23.3 to 33.7 g/L. The final erythritol concentration in these processes ranged from 115 to 148 g/L. In the examined range of nitrogen sources, both the amount and efficiency of erythritol production did not change significantly, while the volumetric production rate of this polyol was definitely the highest (1.00 g/Lh) in the culture with 3.6 g/L of (NH_4_)_2_SO_4_. In this process, 148 g/L of erythritol was obtained with a yield of 0.49 g/g (Figure 3). 

### 2.5. Intensification of the Erythritol Production Process Using the K1UV15 Strain

**Figure 4:**
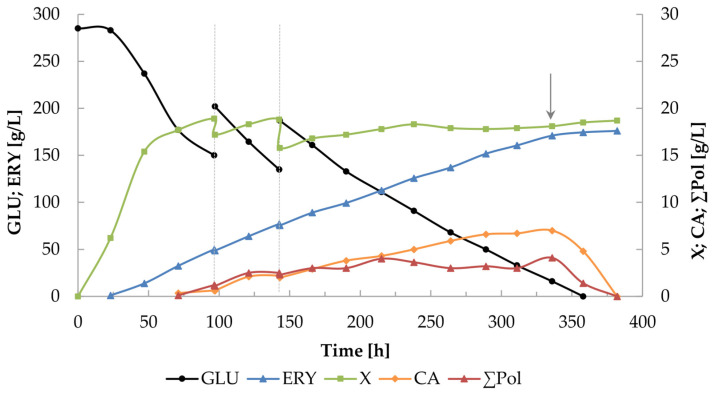
The course of erythritol production using the K1UV15 strain of *Y. lipolytica*, conducted with pulsed glucose feeding to a total concentration of 400 g/L. The arrow indicates the moment of addition of (NH_4_)_2_SO_4_ (3 g/L) and thiamine (B_1_; 200 µg/L). Culture conditions: glucose dosing variant of 250 + 2 × 75 g/L; 2.6 g/L of (NH_4_)_2_SO_4_ at the beginning in the culture medium.

**Figure 5:**
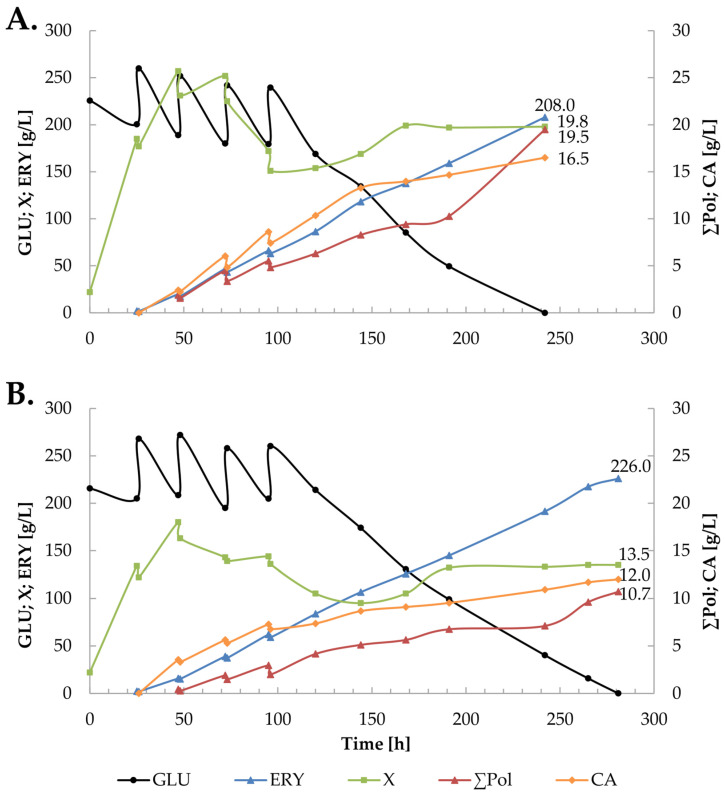
The course of erythritol production using the K1UV15 strain of *Y. lipolytica*, conducted with pulsed glucose feeding to a total concentration of 400 g/L in the medium containing yeast extract (**A**), and in the medium with pure thiamine (**B**). Culture conditions: glucose dosing variant of 150 + 4 × 62.5 g/L; 3.6 g/L of (NH_4_)_2_SO_4_.

In order to improve the parameters of erythritol production from glucose using the *Y. lipolytica* UV15 strain, cultures in which the total glucose concentration was increased to 400 g/L were proposed. Since in the previous experiment, the concentration of erythritol was very similar in the culture with 2.6 and 3.6 g/L of (NH_4_)_2_SO_4_, two cultures were conducted in which both options were examined (Figure 4 and Figure 5). In addition, a culture with 3.6 g/L of (NH_4_)_2_SO_4_ in which the yeast extract was replaced with pure thiamine was performed (Figure 5B).

Figure 4 shows the course of erythritol biosynthesis at a (NH_4_)_2_SO_4_ concentration of 2.6 g/L. The culture was started with 250 g/L of glucose in the medium at the beginning of the process, and the next two portions of glucose, each of 75 g/L, were added successively at 97 and 143 h. The substrate was completely depleted after 358 h of cultivation and the amount of erythritol produced by the yeast reached 174.5 g/L. The yield and volumetric rate of erythritol production calculated at this moment were 0.43 g/g and 0.49 g/Lh, respectively. The concentrations of the by-products—citric acid, mannitol, and arabitol—were 4.8, 0.1, and 1.3 g/L, respectively. In order to improve the selectivity of erythritol production, 3 g/L of (NH_4_)_2_SO_4_ and 200 µg/L of thiamine were added to the culture broth at 336 h of the process. Already on the next day of culture, a significant reduction in the level of by-products and a slight increase in erythritol concentration were noted, and in the 380th hour of the cultivation process, only erythritol was present in the post-culture broth.

Cultures at a higher concentration of (NH_4_)_2_SO_4_ were carried out in two variants: in the first one, the thiamine source was yeast extract (Figure 5A), and in the second, pure thiamine (vitamin B_1_) was used at a concentration of 200 µg/L (Figure 5B). Both cultures were started as batch cultures with an initial glucose concentration of 150 g/L (calculated per final working volume), and the remaining substrate was introduced into the culture in four equal portions (62.5 g/L) every 24 h of the culture. As shown in Figure 5, sugar was utilized more slowly in the culture with pure thiamine, due to the biomass being approximately 6 g/L lower than in the process with the yeast extract. After both cultures, a very high concentration of erythritol was obtained—208 g/L in the process with yeast extract and 226 g/L in the culture with pure thiamine. Compared to the literature reports, this is the highest concentration of erythritol obtained so far from glucose in a biotechnological process using the yeast *Y. lipolytica* (Table 3).

As mentioned above, glucose is not a popular substrate in the biosynthesis of erythritol using the yeast *Y. lipolytica* and there is little information in the literature regarding this process. Wang et al. [28], as a result of a series of genetic modifications, obtained the HCY118 strain with the impaired production of mannitol and d-arabitol, which did not utilize erythritol, could grow at 35 °C, and efficiently produced erythritol at 33 °C. The erythritol production process using this strain was carried out in a medium that contained 300 g/L of glucose and many different components, including 10 g/L of yeast extract and 5 g/L of peptone, and at a high pH of 5.8–6.1. In a 30 m^3^ fermenter after about 78 h of biosynthesis, 196 g/L of erythritol was obtained with a yield and volumetric productivity of 0.65 g/g and 2.51 g/Lh, respectively (Table 3). It is worth noting that these were not only the highest values of the parameters for the production of erythritol from glucose using the *Y. lipolytica* yeast so far but also one of the highest among yeasts of other genera, for which glucose is the most commonly used substrate (Table 3). In studies on erythritol production, various yeasts have been tested, such as *Moniliella*, *Torula*, *Aureobasidium*, *Trichosporon*, *Pseudozyma*, and *Candida*, in batch or fed-batch cultures with a total substrate concentration ranging from 160 to 400 g/L (Table 3). In the study of Saran et al. [45], among various isolates, the best one was *Candida sorbosivorans* SSE-24, for which the optimal composition of the medium for erythritol production was 160 g/L of glucose and 12 g/L of yeast extract. The production of erythritol was successfully increased—in a 30 L bioreactor, with a working volume of 22.5 L, 60.2 g/L of erythritol was produced, with a yield of 0.38 g/g and volumetric productivity of 0.5 g/Lh. Recently, Suwanapetch and Vanichsriratana [44] demonstrated the possibility of using a simple medium containing glucose and soybean flour for the process of erythritol biosynthesis. In a batch culture conducted in a 10 L jar fermenter using *Moniliella* sp. BCC25224, 86.6 g/L of erythritol was produced from 200 g/L of glucose with an efficiency of 0.47 g/g and a volumetric productivity of 0.4 g/Lh. In other studies, in the case of a higher glucose concentration of 300 or 350 g/L using other *Moniliella* sp. strains, a higher erythritol concentration was obtained (90–142 g/L), with slightly lower values of yield but higher volumetric productivity (0.59–1.18 g/Lh) [46,47,49]. In the literature, there are reports of high total glucose concentration (400 g/L) application in industrial cultures of the yeast *Aureobasidium*, as well as *Torula*, *P. tsukubaensis* and *Candida magnoliae* [43,48,50,53]. The *Aureobasidium* sp. SN-G42 mutant, characterized by a lack of foam formation in the bioreactor, was able to produce 175 g/L of erythritol under batch culture conditions with a yield and volumetric production rate of 0.44 g/g and 1.82 g/Lh, respectively. Oh et al. [53] studied the effect of glucose concentration on the production of erythritol using *Torula* sp. yeast in batch culture at a substrate concentration of 400 g/L and obtained very good production parameters. In the same study, the use of a fed-batch culture system allowed them to achieve significant improvements in both erythritol volumetric productivity from 1.43 to 2.26 g/Lh and its production yield from 0.41 to 0.48 g/g (Table 3). Even higher values were obtained in an experiment conducted with *P. tsukubaensis* KN75 in a batch culture with 400 g/L of glucose where 243 g/L of erythritol was obtained [43]. The yield of erythritol production reached as high as 61%, and it should be noted that this was the highest reported value of this parameter by a microorganism so far. In a fed-batch culture with *Candida magnoliae*, increasing the glucose concentration from 300 to 400 g/L resulted in a 2-fold increase in volumetric productivity and a 1.8-fold increase in the yield of erythritol production [48]. However, in both of these processes, very high concentrations of by-products were observed, mainly citric and butyric acids, the total concentration of which was approximately 120 and 137 g/L, respectively, in the process with 300 and 400 g/L of glucose [48].

## 3. Materials and Methods

### 3.1. Microorganisms

The study examined eight *Yarrowia lipolytica* yeast strains obtained by the UV mutagenization of the *Y. lipolytica* Wratislavia K1 strain. The parental strain *Y. lipolytica* Wratislavia K1 used in this study was obtained from the culture collection of the Department of Biotechnology and Food Microbiology, Wroclaw University of Environmental and Life Sciences, Poland. 

### 3.2. Procedure of Mutagenization

Under sterile conditions, the yeast biomass from the YM agar slant was washed off with a physiological solution of NaCl (0.9%) and the density of the obtained suspension (3.46 × 10^7^ cells/mL) was determined using a Thoma counting chamber (Figure 1). The volume of 10 mL of cell suspension was transferred to each sterile crystallizer with a diameter of 0.05 m and containing a magnetic stirring bar. The crystallizers were placed on a magnetic stirrer (Big Squid, IKA, Berlin, Germany) under a UV lamp (UV Lamp 4, Camag, Muttenz, Switzerland) with a wavelength of 254 nm and a power of 14 W. The distance from the radiation source to the suspension was 0.25 m. The suspensions were continuously stirred (800 rpm) and exposed to irradiation for 20 and 40 s and 1, 3, 5, 10, 15, 30, and 45 min, then poured into sterile falcons and placed in the darkroom for one hour to avoid photoreactivation. Serial decimal dilutions of each sample were inoculated into Petri dishes with YM-agar medium, which were then incubated at 30 °C in a laboratory incubator (Memmert GmbH, Schwabach, Germany). After 3 and 6 days of incubation, the morphology was assessed, the number of colonies grown was counted, and the survival rate was calculated.

### 3.3. Media

The YM-agar medium used for UV mutant selection contained the following [g/L]: glucose—10.0; yeast extract—3.0; malt extract—3.0; bactopeptone—5.0; and agar—20.0, dissolved in distilled water. The medium used for the preparation of inoculum for shake-flask cultures contained the following [g/L]: glucose—50.0; yeast extract—3.0; malt extract—3.0; and bactopeptone—5.0, dissolved in distilled water. The composition of the shake-flask medium for assessing the ability to grow and utilize erythritol was the following [g/L]: erythritol—43.0; (NH_4_)_2_SO_4_—2.5; MgSO_4_·7H_2_O—1.0; KH_2_PO_4_—0.22; and yeast extract—1.0, dissolved in distilled water. The medium for screening performed in shake-flask cultures contained the following [g/L]: glucose—100.0; (NH_4_)_2_SO_4_—2.5; MgSO_4_·7H_2_O—1.0; KH_2_PO_4_—0.22; yeast extract—1.0; NaCl—26.4; and CaCO_3_—30.0, distilled water up to 1 L. The medium used for the preparation of inoculum for the bioreactor culture contained the following [g/L]: glucose—40; peptone—2.0; and yeast extract—3.0, distilled water up to 1 L. The production medium for bioreactor cultures had the following composition [g/L]: glucose 300 or 400 (with dosing strategies marked on documents); (NH_4_)_2_SO_4_—2.6/3.1/3.6/4.1; MgSO_4_·7H_2_O—1.0; KH_2_PO_4_—0.25; and yeast extract—1.0 (or thiamine—200 µg/L; in case of culture shown in Figure 5B), distilled water up to 1 L, pH 3.0. The media were sterilized at 121 °C for 20 min. All the chemicals used in the study were of analytical purity (Sigma-Aldrich, Sternheim, Germany).

### 3.4. Culture Conditions

Inoculation cultures were carried out on a rotary shaker (Certomat IS, Sartorius Stedim Biotech GmbH, Göttingen, Germany) in conical flasks with a volume of 300 mL containing 50 mL of inoculation medium, at a temperature of 29.0 °C and a speed of 140 rpm for a period of 72 h. Shake-flask cultures (examination of growth in medium with erythritol and screening in glucose-based medium) were carried out in triplicate in 300 mL conical flasks containing 30 mL of the appropriate medium, for 72 or 96 h, under the conditions as described above. Bioreactor production cultures were carried out in duplicate in a 5 L Biostat B Plus fermenter (Sartorius, Germany) with a working volume of 2 L. During the culture, the pH was automatically adjusted to the level of 3.0–3.1 using 20% NaOH, the temperature was set at 29.0 °C, the agitation was 700 rpm, and the aeration of 0.8 vvm was used. 

### 3.5. Analytical Methods

Biomass (X) in the culture sample (10 mL) was determined using the gravimetric method after membrane filtration (pore size of Ø = 0.22 μm; Merck, Darmstadt, Germany) and drying at 105 °C (MAC110/NH, Radwag, Poland). The concentration of glucose (GLU) and the products, citric acid (CA), erythritol (ERY), mannitol, and arabitol (ƩPol), was determined by the HPLC method [27] using Carbohydrate H^+^ Column (Thermo Scientific, Waltham, MA, USA) coupled to a UV detector (λ = 210 nm; Dionex, Sunnyvale, CA, USA) and a refractive index detector (Shodex, Ogimachi, Japan). The column was eluted with 25 mM of trifluoroacetic acid at 65 °C and a flow rate of 0.6 mL/min. The statistical analysis was performed using a one-way analysis of variance (Statistica 13.0 software; StatSoft, Tulsa, OK, USA). The significant differences in the data were compared using Duncan’s multiple range test (*p* ≤ 0.05).

## 4. Conclusions

In this study, a promising erythritol-producing strain, *Y. lipolytica* K1UV15, was obtained by UV mutagenesis. The strain exhibited good technological features such as a lack of foam formation, a low production of by-products, the inability to utilize erythritol, and good parameters of erythritol biosynthesis from glucose at low pH, which may be a good alternative to processes with glycerol. The breeding system investigated in this work with a glucose concentration increased to 400 g/L and at an increased level of biomass allowed us to obtain 226 g/L of erythritol with a yield close to the theoretical value and with a production volumetric rate of up to 0.86 g/Lh.

## Figures and Tables

**Table 1:** Characteristics of UV mutants of *Y. lipolytica* in shake-flask cultures.

Strain	Substrate Consumption Rate		Erythritol Production from Glucose		
	q_S(ERY)_	q_S(GLU)_	ERY *	Y_ERY_	q_ERY_
	[mg/Lh]	[g/Lh]	[g/L]	[g/g]	[mg/gh]
K1UV1	0.00 ± 0.00 ^f^ **	1.022 ± 0.034 ^a^	24.9 ± 1.3 ^c^	0.25 ± 0.02 ^bcd^	31.25 ± 2.55 ^a^
K1UV3	69.4 ± 6.4 ^d^	0.881 ± 0.040 ^c^	22.1 ± 1.4 ^e^	0.26 ± 0.02 ^bc^	17.17 ± 1.18 ^d^
K1UV11	313.9 ± 17.5 ^a^	0.906 ± 0.046 ^bc^	23.0 ± 0.6 ^d^	0.26 ± 0.00 ^bc^	26.62 ± 4.36 ^c^
K1UV15	no growth	1.041 ± 0.040 ^a^	26.5 ± 1.9 ^b^	0.27 ± 0.02 ^b^	28.75 ± 2.95 ^ab^
K1UV16	125.0 ± 14.0 ^b^	0.893 ± 0.026 ^bc^	20.3 ± 0.8 ^f^	0.24 ± 0.02 ^d^	14.09 ± 0.93 ^d^
K1UV17	90.3 ± 3.8 ^c^	0.932 ± 0.020 ^bc^	26.3 ± 1.8 ^b^	0.29 ± 0.02 ^a^	29.14 ± 1.84 ^a^
K1UV18	34.7 ± 1.8 ^e^	1.030 ± 0.032 ^a^	25.3 ± 0.6 ^c^	0.26 ± 0.00 ^bc^	25.09 ± 1.88 ^bc^
K1UV20	95.8 ± 5.6 ^c^	0.947 ± 0.036 ^b^	27.4 ± 2.1 ^a^	0.30 ± 0.02 ^a^	29.42 ± 1.79 ^a^
Wratislavia K1	0.00 ± 0.00 ^f^ **	0.897 ± 0.026 ^bc^	21.6 ± 0.4 ^e^	0.25 ± 0.01 ^cd^	22.95 ± 1.07 ^c^

q_S_—substrate consumption rate of erythritol (ERY) and glucose (GLU); Y_ERY_—yield of erythritol in terms of substrate consumed; q_ERY_—erythritol specific production rate; the mean values and standard deviations were calculated from the data obtained in three different replications. * Values in the same column marked with different letters differ significantly at *p* ≤ 0.05. ** No cell growth.

**Table 2:** Technological characteristics of UV mutants of *Y. lipolytica* in bioreactor culture.

Strain	Time [h]	ERY * [g/L]	Y_ERY_ [g/g]	Q_ERY_ [g/Lh]	q_ERY_ [g/gh]	S [%]	Foam **
K1UV1	196	127.0 ^c^	0.42 ± 0.02 ^bc^	0.65 ± 0.01 ^d^	0.026 ± 0.001 ^d^	83	+
K1UV3	196	134.6 ^b^	0.45 ± 0.02 ^ab^	0.69 ± 0.01 ^c^	0.036 ± 0.003 ^b^	84	+
K1UV11	145	106.4 ^e^	0.35 ± 0.01 ^ef^	0.73 ± 0.00 ^b^	0.031 ± 0.002 ^c^	78	−
K1UV15	183	145.0 ^a^	0.48 ± 0.03 ^a^	0.79 ± 0.09 ^a^	0.034 ± 0.002 ^bc^	88	−
K1UV16	196	98.5 ^f^	0.33 ± 0.01 ^f^	0.50 ± 0.00 ^e^	0.025 ± 0.003 ^d^	74	+
K1UV17	168	124.1 ^c^	0.41 ± 0.02 ^cd^	0.74 ± 0.01 ^b^	0.035 ± 0.003 ^bc^	77	+
K1UV18	250	115.0 ^d^	0.38 ± 0.02 ^de^	0.46 ± 0.01 ^f^	0.020 ± 0.001 ^e^	86	+
K1UV20	167	124.0 ^c^	0.41 ± 0.02 ^cd^	0.74 ± 0.01 ^b^	0.042 ± 0.003 ^a^	86	+
Wratislavia K1	222	112.0 ^d^	0.37 ± 0.02 ^ef^	0.50 ± 0.01 ^e^	0.022 ± 0.001 ^ed^	82	−

Culture conditions: 250 + 50 g/L glucose dosing variant; 2.6 g/L of (NH_4_)_2_SO_4_. Y_ERY_—yield of erythritol in terms of substrate consumed; Q_ERY_—erythritol volumetric production rate [g/Lh]; q_ERY_—erythritol specific production rate; S—selectivity of KGA (expressed as % of erythritol in the pool of products); * values in the same column marked with different letters differ significantly at *p* ≤ 0.05. ** Formation (+) or lack of foam formation (−) in the bioreactor during the production process.

**Table 3:** Comparison of erythritol production from glucose by different yeast-like fungi.

Microorganism	Cultivation System; Tank Volume; Working Volume	GLU [g/L]; Nitrogen Source [g/L]; pH		ERY [g/L]	Q_ERY_ [g/L/h]	Y_ERY_ [g/g]	Ref.
*Candida sorbosivorans SSE-24*	Batch; 30 L; 22.5 L	160; yeast extract—12; 5.0		60.2	0.5	0.38	[45]
*Moniliella* sp. BCC25224	Batch, stirred tank, 10 L; not specified	200; soybean flour—13; 6.0		86.6	0.4	0.47	[44]
*Moniliella* sp. 440	Batch, stirred tank; 5 L; 2 L	300; yeast extract—10; 5.3		116	0.81	0.39	[46]
*Moniliella tomentosa* var. *pollinis*	Batch, stirred tank; 30 L; 20 L	352; 2% CSL—20 + 0.1% urea—1; 2.5–3.4		90.0	0.59	0.35	[47]
	Fed-batch			170	1.62	0.38	
*Candida magnoliae*	Fed-batch; 3.3 L;	300 in the production stage; yeast extract; 4.5→3.2		84	1.4	0.23	[48]
		400 in the production stage; yeast extract; 4.5→3.2		187	2.8	0.41	
*Moniliella* sp. N61188-12 (NTG mutant)	Fed-batch, 2000 L; 800 L	400; yeast extract—10; 7.0		237.8	1.98	0.59	[49]
*Aureobasidium* sp. SN-G42	Batch; 5 L;	400		175	1.82	0.44	[50]
*Trichosporon* sp.	Batch; 5 L; not specified	220; corn steep liquor—45; 3.5		not specified	1.51	0.4	[51]
		300; corn steep liquor—45; 3.5		not specified	1.23	0.46	
	Fed-batch; 5 L; 2.5→3.25 L	220; corn steep liquor—45; 3.5		not specified	1.54	0.45	
*Torula* sp.	Batch; 5 L; 3 L	400; yeast extract—20; initial 5.5		155	1.08	0.38	[52]
				182 ^1^	1.34	0.45	
*Torula* sp.	Batch, stirred tank, 5 L; 3 L	400; yeast extract—20; 5.5		193	1.43	0.41	[53]
	Fed-batch, stirred tank; 5 L; 2.4→3 L	400; yeast extract—20; 5.5		192	2.26	0.48	
*P. tsukubaensis*	Batch; 50,000 L; 6000 L	400; corn steep powder—15; 5.5		243	1.65	0.61	[43]
	Fed-batch; 50,000 L; 6000 L	400; corn steep powder—15; 5.5		241	2.84	0.60	
*Y. lipolytica* CGMCC7326 (wild type)	Batch; 30,000 L; 22,500 L	300; yeast extract—10 + peptone—5 + ammonium citrate—3.5 + (NH_4_)_2_HPO_4_—0.1; 5.8–6.1		165	1.57	0.55	[28]
*Y. lipolytica* HCY118 (transformant)				196 ^2^	2.51	0.65	
*Y. lipolytica YALI-hsp90*	Batch, stirred tank, 5 L; 3 L	300; yeast extract powder—8 + tryptone—2 + diammonium hydrogen phosphate—2 + ammonium citrate—2; 6.5–7.0		76.4 ^2a^	0.85	0.25	[29]
*Y. lipolytica* Wratislavia K1	Fed-batch; stirred tank, 5 L; 2 L;	240; (NH_4_)_2_SO_4_—4.6; 3.0		72.2 ^3^	1.02	0.43	[27]
*Y. lipolytica* Wratislavia K1	Fed-batch; stirred tank, 5 L; 2 L;	300; NH_4_Cl—3 + yeast extract—1; 3.0		23 ^3^	0.09	0.14	[20]
*Y. lipolytica* Wratislavia K1	Batch; stirred tank, 5 L; 2 L;	300	2.6 g/L (NH_4_)_2_SO_4_	71.3	0.33	0.37	This study
	Fed-batch, stirred tank, 5 L; 2 L;	300 (250 + 50)		97.5	0.5	0.39	
*Y. lipolytica* K1UV15		300 (250 + 50)		145	0.79	0.48	
		400 (250 + 2 × 75)		174.5	0.49	0.43	
		400 (150 + 4 × 62.5; YE)	3.6 g/L (NH_4_)_2_SO_4_	208	0.86	0.52	
		400 (150 + 4 × 62.5; B_1_)		226	0.50	0. 565	

^1^ Supplemented with phytic acid; ^2^ fermentation temperatures of 33 °C and ^2a^ 34 °C; ^3^ glucose solution was continuously fed into the fermenter.

## Data Availability

The data are contained within the article.

## Supplementary Materials

The following supporting information can be downloaded at: https://www.mdpi.com/article/10.3390/molecules29102187/s1, Table S1. Characteristics of UV mutants of *Y. lipolytica* on YM-agar plates.
