# Supplementary material for: Enhanced Production of Erythritol from Glucose by the Newly Obtained UV Mutant Yarrowia lipolytica K1UV15
Source: Molecules. 2024 May 8;29(10):2187. doi: 10.3390/molecules29102187 (PMC11124037; doi:10.3390/molecules29102187)
Supplement: Supplementary file 1 [file molecules-29-02187-s001.zip › molecules-2984382-supplementary.pdf]

Table S1. Characteristics of UV mutants of *Y. lipolytica* on YM-agar plates.

UV-exposure time [min]	Strain	Growth on YM-agar	
		Colony size [mm]	Colony surface
15	K1UV1	7.1	smooth
15	K1UV3	6.5	smooth
30	K1UV11	9.4	smooth
5	K1UV15	6.9	smooth
10	K1UV16	8.2	rough
10	K1UV17	5.2	rough
10	K1UV18	8.7	smooth
10	K1UV20	9.2	smooth
0	Wratislavia K1	8.0	smooth
